# Supplementary material for: Bet hedging in a unicellular microalga
Source: Nat Commun. 2024 Mar 7;15:2063. doi: 10.1038/s41467-024-46297-6 (PMC10920660; doi:10.1038/s41467-024-46297-6)
Supplement: Supplementary file 1 — Supplementary Information [file 41467_2024_46297_MOESM1_ESM.pdf]

Supplementary information for:

Bet hedging in a unicellular microalga

Si Tang[1], Yaqing Liu[1], Jianming Zhu[1], Xueyu Cheng[1], Lu Liu[1], Katrin Hammerschmidt[2,*], Jin Zhou[1,*], Zhonghua Cai[1,3,*]

[1] Shenzhen Public Platform for Screening and Application of Marine Microbial Resources, Tsinghua Shenzhen International Graduate School, Shenzhen, 518055, Guangdong Province, PR China.

[2] Institute of General Microbiology, Kiel University, Kiel, Germany.

[3] Technology Innovation Center for Marine Ecology and Human Factor Assessment of Natural Resources Ministry, Tsinghua Shenzhen International Graduate School, Shenzhen, 518055, Guangdong Province, PR China.

*Correspondence: katrinhammerschmidt@googlemail.com (K.H.), zhou.jin@sz.tsinghua.edu.cn (J.Z.), caizh@sz.tsinghua.edu.cn (ZH.C.)

Includes:

Supplementary figures 1-4

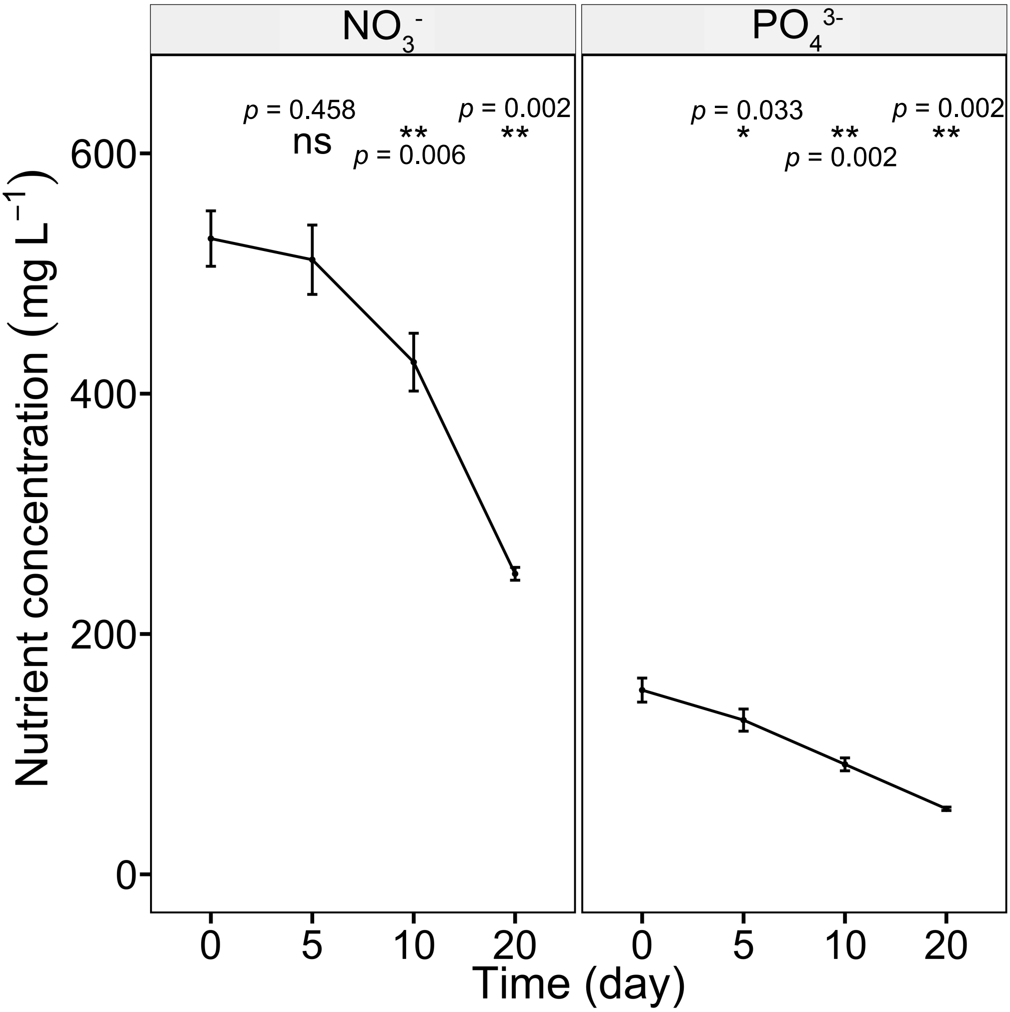

Supplementary Fig. 1. Nutrient dynamics over time. The concentration of dissolved NO_3^- and PO_4^{3-} was quantified over the period of 20 days. The graphs show the mean ± standard deviation (n = 3). Corresponding significance markers represent statistical significance calculated by two-tailed *t* test between data obtained from different time points and that of day 0. Significance: ns (no significance), *($p < 0.05$), **($p < 0.01$), ***($p < 0.001$), ****($p < 0.0001$). Source data are provided as a Source Data file.

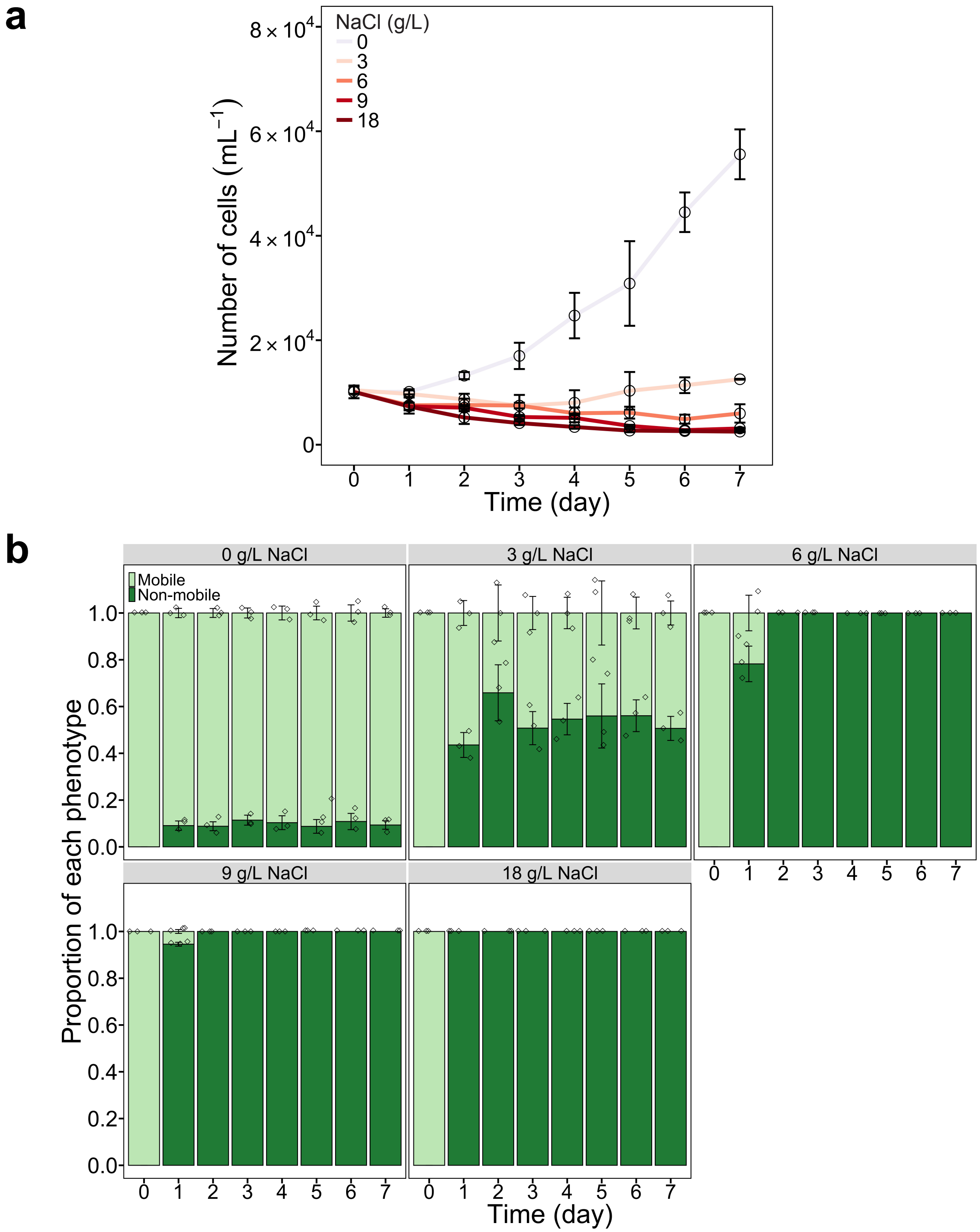

Supplementary Fig. 2. Performance of *H. pluvialis* populations under NaCl stress. a Growth curves of *H. pluvialis* populations exposed to different levels of NaCl stress over a 7-day period. The graph shows the mean ± standard deviation (n = 3). **b** Change in the proportion of mobile (light green) and non-mobile phenotype (dark green) in *H. pluvialis* populations over a period of 7 days. The graphs show the mean ± standard deviation (n = 3). Source data are provided as a Source Data file.

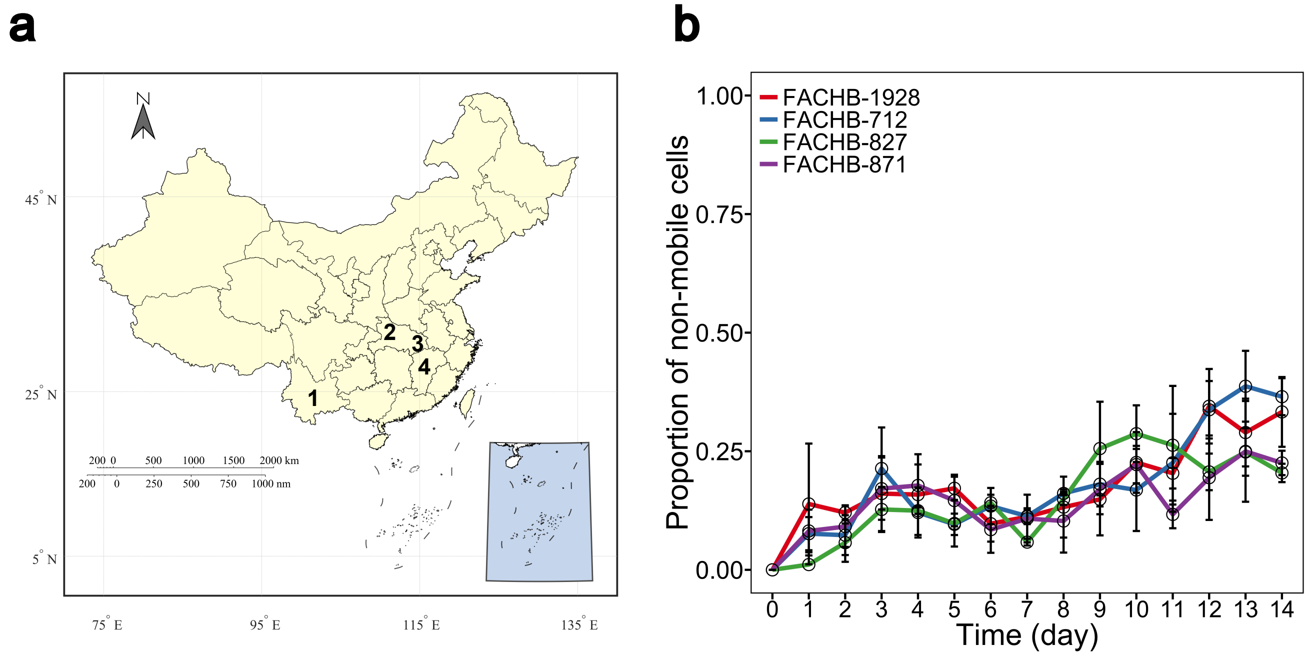

Supplementary Fig. 3. Phenotypic diversification of four strains of *H. pluvialis* into mobile and non-mobile cells. a Sampling sites of the different *H. pluvialis* strains. 1, FACHB-1928, Yunnan province, China; 2, FACHB-712, Hubei province, China; 3, FACHB-827, Hubei province, China; 4, FACHB-871, Jiangxi province, China. **b** The proportion of non-mobile cells of four *H. pluvialis* strains over a 14-day period. Note that FACHB-712 (blue color) is the focal organism used in this study. FACHB-1928 (pink), FACHB-712 (blue), FACHB-827 (green), FACHB-871 (purple). The graph shows the mean ± standard deviation (n = 3). Source data are provided as a Source Data file.

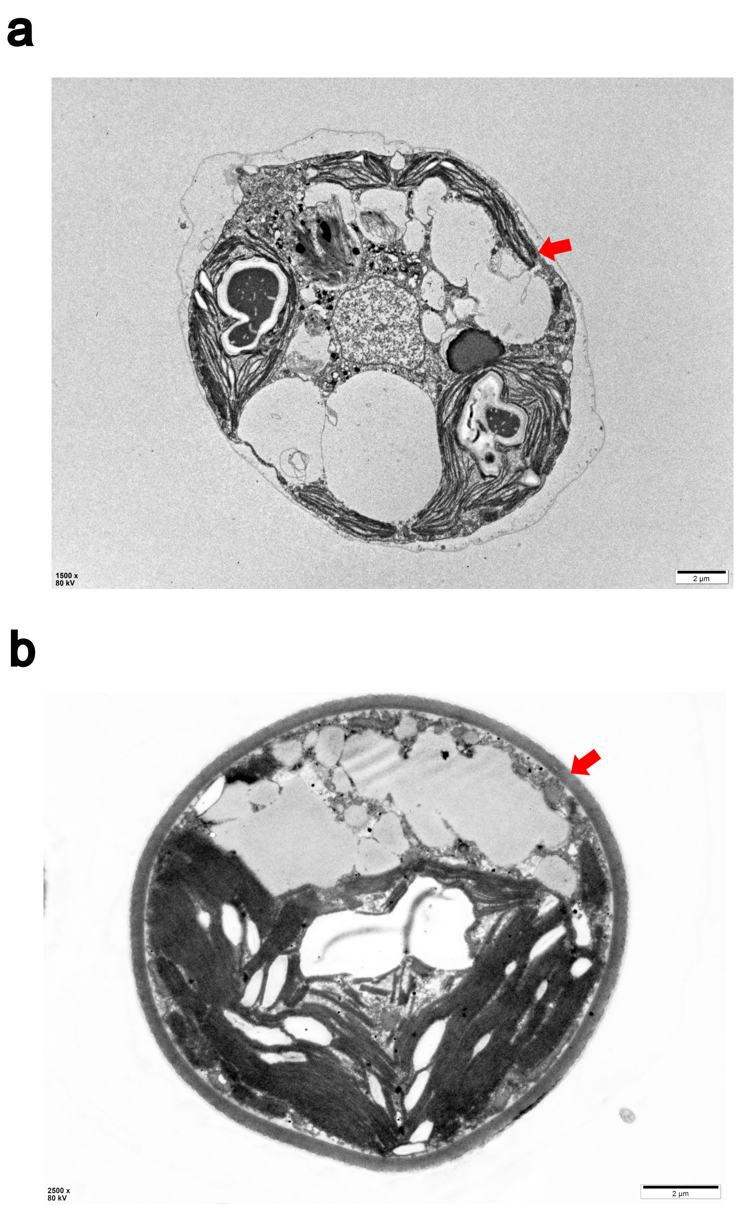

Supplementary Fig. 4. TEM of cell wall structure of both cell types in *H. pluvialis*. TEM of cell wall structure of a mobile cell (**a**) and a non-mobile cell (**b**). Cell wall is indicated by the red arrow. Non-mobile cells developed a significantly thicker cell wall relative to that of mobile cells.TEM was conducted twice and five cells were checked each time, similar results were observed. Scale bar: 2 µm.
