# Supplementary material for: Bet hedging in a unicellular microalga
Source: Nat Commun. 2024 Mar 7;15:2063. doi: 10.1038/s41467-024-46297-6 (PMC10920660; doi:10.1038/s41467-024-46297-6)
Supplement: Supplementary file 3 — Reporting Summary [file 41467_2024_46297_MOESM3_ESM.pdf]

Corresponding author(s): Katrin Hammerschmidt, Jin Zhou; Zhonghua Cai

Last updated by author(s): 2024.02.07

Reporting Summary

Nature Portfolio wishes to improve the reproducibility of the work that we publish. This form provides structure for consistency and transparency in reporting. For further information on Nature Portfolio policies, see our Editorial Policies and the Editorial Policy Checklist.

Statistics

For all statistical analyses, confirm that the following items are present in the figure legend, table legend, main text, or Methods section.

n/a	Confirmed	
☐	☒	The exact sample size (*n*) for each experimental group/condition, given as a discrete number and unit of measurement
☐	☒	A statement on whether measurements were taken from distinct samples or whether the same sample was measured repeatedly
☐	☒	The statistical test(s) used AND whether they are one- or two-sided<br>*Only common tests should be described solely by name; describe more complex techniques in the Methods section.*
☒	☐	A description of all covariates tested
☒	☐	A description of any assumptions or corrections, such as tests of normality and adjustment for multiple comparisons
☐	☒	A full description of the statistical parameters including central tendency (e.g. means) or other basic estimates (e.g. regression coefficient) AND variation (e.g. standard deviation) or associated estimates of uncertainty (e.g. confidence intervals)
☐	☒	For null hypothesis testing, the test statistic (e.g. *F*, *t*, *r*) with confidence intervals, effect sizes, degrees of freedom and *P* value noted<br>*Give P values as exact values whenever suitable.*
☒	☐	For Bayesian analysis, information on the choice of priors and Markov chain Monte Carlo settings
☒	☐	For hierarchical and complex designs, identification of the appropriate level for tests and full reporting of outcomes
☒	☐	Estimates of effect sizes (e.g. Cohen's *d*, Pearson's *r*), indicating how they were calculated

Our web collection on statistics for biologists contains articles on many of the points above.

Software and code

Policy information about availability of computer code

Data collection	All scripts for data visualisation are available at https://github.com/SiTANG1990/Microalgal-bet-hedging/tree/v1.0.0 (https://doi.org/10.5281/zenodo.10578478).
Data analysis	All statistical analyses were performed in R v4.1.1. Statistical significance for physiological measurements and nutrient dynamics was calculated by Welch's t test for pairwise comparisons of two treatments (p value < 0.05). A one-way ANOVA with Tukey's HSD post-hoc analysis (p value < 0.05) was conducted for all tested populations of growth and survival assays. Differential gene expression (DGE) was conducted with DESeq2 v1.32.0.

For manuscripts utilizing custom algorithms or software that are central to the research but not yet described in published literature, software must be made available to editors and reviewers. We strongly encourage code deposition in a community repository (e.g. GitHub). See the Nature Portfolio guidelines for submitting code & software for further information.

Data

Policy information about availability of data

All manuscripts must include a data availability statement. This statement should provide the following information, where applicable:

- Accession codes, unique identifiers, or web links for publicly available datasets
- A description of any restrictions on data availability
- For clinical datasets or third party data, please ensure that the statement adheres to our policy

The data of this study are available within the article. Raw RNAseq reads for differential gene expression analyses have been submitted to NCBI's SRA database (http://www.ncbi.nlm.nih.gov) under BioProject PRJNA940855. The KEGG database (https://www.kegg.jp/) was used for functional enrichment analyses. Source data are provided with this paper.

Research involving human participants, their data, or biological material

Policy information about studies with human participants or human data. See also policy information about sex, gender (identity/presentation), and sexual orientation and race, ethnicity and racism.

Reporting on sex and gender

There is no such information in this study.

Reporting on race, ethnicity, or other socially relevant groupings

There is no such information in this study.

Population characteristics

There is no such information in this study.

Recruitment

There is no such information in this study.

Ethics oversight

There is no such information in this study.

Note that full information on the approval of the study protocol must also be provided in the manuscript.

Field-specific reporting

Please select the one below that is the best fit for your research. If you are not sure, read the appropriate sections before making your selection.

☐ Life sciences ☐ Behavioural & social sciences ☒ Ecological, evolutionary & environmental sciences

For a reference copy of the document with all sections, see nature.com/documents/nr-reporting-summary-flat.pdf

Ecological, evolutionary & environmental sciences study design

All studies must disclose on these points even when the disclosure is negative.

Study description

In this study, bet hedging was proposed to explain the phenotypic diversification of an isogenic population of unicellular microalga Haematococcus pluvialis. Together with imaging with the light microscope and transmission electron microscope, we conducted five growth experiments, stress tests, quantification of physiological parameters, and transcriptomics to support our proposal. In these quantification experiments, all treatments, i.e., mobile cells and non-mobile cells, had three replicates, and for transcriptomic analysis, triplicate randomly-sampled cultures of two treatments were investigated.

Research sample

The research sample studied in this study was a widely distributed, buoyant unicellular biflagellate freshwater microalga Haematococcus pluvialis (Chlorophyceae, Volvocales). The strain FACHB-712 was purchased from the Freshwater Algae Culture Collection at the Institute of Hydrobiology, Wuhan, China. The strain was chosen as the model organism due to the observation of its early phenotypic diversification, which fits the bet hedging theory. During the experiments, cells from exponential phase (10 days old) since new inoculation were collected for further analyses. Mobile and non-mobile samples are defined by their mobility, representing various physiological and metabolic activities.

Sampling strategy

No statistical methods were used to predetermine sample size in this study. In general, n = 3 was chosen as the replicate number based on previous experience and standards in the field (https://doi.org/10.1038/s41396-022-01307-7). For cell size quantification, at least 300 cells, quantitatively sufficient for cell size estimation, were measured for each treatment.

Data collection

For imaging with the light microscope, 100 µL of respective microalgal culture was checked and imaged under the microscope by Si Tang. TEM was conducted by Jiangsu Meimian Industrial Co., Ltd. Transcriptomics was performed by Shanghai Majorbio Bio-pharm Technology Co., Ltd. (Shanghai, China). Yaqing Liu recorded the data from the quantification of physiological parameters, and Si Tang recorded the data from all other experiments, these data were collected and recorded manually with pen and paper.

Timing and spatial scale

We first conducted a pre-experiment, and based on the pre-results, we designed these formal experiments. A series of formal experiments were conducted in October & November 2022 and November 2023. In detail, phenotypic diversification recording was

investigated from 05.10.2022 to 25.10.2022. During this period, data collection, including imaging, cell size quantification, quantification of physiological parameters, stress tests, sample preparation for transcriptomics, was conducted. Phenotypic rediversification experiment was performed in November, 2022. Tests for different H. pluvialis strains and NaCl stress test were conducted in November, 2023. For all growth experiment, data were collected daily to get a precise evaluation of population performance. For the phenotypic diversification experiment , the experiment lasted 20 days until almost all cells turned from mobile to non-mobile. Simultaneously, the residual nutrients experiment was conducted and the residual nutrients were quantified every five days as such a setup can allow us to have a general understanding of the nutrient usage trend. The rediversification experiment stopped on day 10 when we observed phenotypic diversification again. For the stress test, we collected the data on day 11 as we observed significant population changes based on daily observation (population changes) and results from preliminary experiments. There was no gap between collection periods and no spatial scale from which the data were taken.

Data exclusions	No data were excluded from the analysis.
Reproducibility	In general, all experiments were done in triplicate, and all attempts of replication were successful and showed similar results. For our central observation (the early phenotypic diversification), we conducted two pre-experiments before collecting formal data, and all trials resulted in similar results. For physiological and transcriptomic data, their patterns were consistent with previously known results.
Randomization	Since our model organism is photoautotrophic, during experiments, the positions of culture flasks were randomly mixed daily to reduce possible differences in growth due to light availability. Microalgal cell cultures were gently mixed for randomization for all experiments in this study before pipetting out for further analysis.
Blinding	The persons quantifying cellular parameters and TEM imaging were unaware of the sample identity. Numerical data have been analyzed double-masked, resulting in the same results as in the non-blinded analysis.

Did the study involve field work? ☐ Yes ☒ No

Reporting for specific materials, systems and methods

We require information from authors about some types of materials, experimental systems and methods used in many studies. Here, indicate whether each material, system or method listed is relevant to your study. If you are not sure if a list item applies to your research, read the appropriate section before selecting a response.

Materials & experimental systems

n/a	Involved in the study
☒	☐ Antibodies
☒	☐ Eukaryotic cell lines
☒	☐ Palaeontology and archaeology
☒	☐ Animals and other organisms
☒	☐ Clinical data
☒	☐ Dual use research of concern
☒	☐ Plants

Methods

n/a	Involved in the study
☒	☐ ChIP-seq
☒	☐ Flow cytometry
☒	☐ MRI-based neuroimaging

Plants

Seed stocks	Haematococcus pluvialis (FACHB-712, Freshwater Algae Culture Collection at the Institute of Hydrobiology, Wuhan, China)
Novel plant genotypes	There is no such information in this study.
Authentication	The model organism used in this study was purchased from a commercial microalgae culture collection and was authenticated for scientific research. There was no novel genotype generated in this study.
